# Hsa_circYARS interacts with miR-29a-3p to up-regulate IREB2 and promote laryngeal squamous cell carcinoma progression

**DOI:** 10.1007/s12672-024-01198-4

**Published:** 2024-09-03

**Authors:** Zizhao Guo, Yuxia Zhao, Naicai Guo, Meng Xu, Xiaolei Wang

**Affiliations:** 1 Department of Head and Neck Surgery, National Cancer Center, National Clinical Research Center for Cancer; Cancer Hospital, Chinese Academy of Medical Sciences and Peking Union Medical College, No. 17, Panjiayuan Nanli, Chaoyang District, Beijing City, 100021 China; 2 Department of Radiology, National Cancer Center, National Clinical Research Center for Cancer; Cancer Hospital, Chinese Academy of Medical Sciences and Peking Union Medical College, Beijing City, 100021 China; 3 Department of Radiology, Beijing Hospital, National Center of Gerontology, Beijing City, 100730 China; 4 Department of Head and Neck Surgery, Sir Run Run Shaw Hospital, Zhejiang University School of Medicine, Hangzhou, China; 5 Department of Radiology, Beijing Hospital, National Center of Gerontology, No. 1, Dongdan Dahua Road, Beijing City, 100730 Dongcheng District China

**Keywords:** circYARS, miR-29a-3p, IREB2, Laryngeal squamous cell carcinoma

## Abstract

**Objective:**

This study was to investigate the carcinogenic capacity of circYARS in laryngeal squamous cell carcinoma (LSCC) and to reveal its potential mechanism as a competitive endogenous RNA.

**Methods:**

The differentially expressed circRNA and mRNA in LSCC were detected by RT-qPCR. Dual luciferase reporter assay and RIP were conducted to test the interaction between circYARS, miR-29a-3p, and IREB2. The functional effects of these molecules were investigated by CCK-8, flow cytometry, colony formation assay, Transwell, Western blot, and xenotransplantation mouse models.

**Results:**

In LSCC tissues and cell lines, circYARS and IREB2 levels were enhanced, while miR-29a-3p level was lowered. Depleting circYARS led to decreased IREB2 by promoting miR-29a-3p expression. As a result of miR-29a-3p enhancement or circYARS silence, the proliferative, migratory, and invasion of cancer cells were suppressed and apoptosis was stimulated.

**Conclusion:**

circYARS is involved in the tumorigenicity and progression of LSCC through the miR-29a-3p/IREB2 axis, providing strategies and targets for therapeutic intervention of LSCC.

## Introduction

As a malignant tumor of the head and neck, laryngeal squamous cell carcinoma (LSCC) develops from the laryngeal epithelium. An estimated one-third of all head and neck cancers occur in the larynx, making it the sixth most common form of cancer worldwide [1, 2]. Although the incidence of LSCC has decreased, as many patients are diagnosed in the later stages, they do not receive adequate treatment, resulting in a declining five-year survival rate [3, 4]. Most patients ultimately die from cancer recurrence and metastasis. Therefore, a better understanding of the pathogenesis of LSCC will provide new diagnostic and prognostic indicators for the treatment of this disease.

Circular RNAs (circRNAs) are novel regulatory RNAs characterized by the presence of covalently closed loops and the absence of free 5′ to 3′ ends [5]. There is now increasing evidence that aberrant circRNA expression is closely related to the pathogenesis of LSCC, with effects on gene expression, cell proliferation, and regulation of migration, thereby altering invasion, apoptotic capacity, and cell cycle progression [6]. The relationship between circRNAs and laryngeal cancer can be broadly classified into two scenarios, including down-regulation of tumor suppressor circRNAs and up-regulation of tumor-metastatic circRNAs. circRNAs are potential targets for the diagnosis and treatment of LSCC [7]. It has been reported that circPARD3 inhibits autophagy through PRKCI/Akt/mTOR signaling and promotes chemoresistance in LSCC cells during cancer progression [8]. CircRNAs play a variety of cellular functions in LSCC progression through different mechanisms including specific binding to miRNAs, thereby eliminating the inhibitory effect of miRNAs on target genes and up-regulating the expression level of target genes, i.e., acting as competitive endogenous RNAs (ceRNAs) [9–11]. In addition, circRNAs may serve as biomarkers in the diagnosis or prognosis of various cancers due to their tissue-specific expression patterns and remarkable stability. Wu et al. screened differentially expressed circRNAs by RNA sequencing and found that circYARS is highly expressed in LSCC [12], but its role, regulatory mechanism and expression pattern remain largely unknown.

MiRNAs are single-stranded non-coding RNA molecules, ranging in length from 19–25 nucleotides. Human pathophysiology depends heavily on miRNAs, which regulate signal molecules to be involved in proliferation, differentiation, and apoptosis [13, 14]. miRNAs have been recognized as markers of early diagnosis and prognosis in patients with LSCC [15–17]. It has been indicated that regulating miRNA expression is a promising method to control tumor progression [18]. miR-29a-3p has recently received a lot of attention for its alterations in human cancers [19–21]. miR-29a-3p is aberrantly expressed in oral squamous cell carcinoma cells; inhibition of cell viability, proliferation, and migration and increase in apoptosis correlate with up-regulation of miR-29a-3p [22].

Iron-responsive element binding protein 2 (IREB2) is overexpressed in breast cancer, and IREB knockout can delay the growth of breast tumors [23]. However, the relationship between the IREB2 gene and LSCC has not been studied.

circYARS is aberrantly expressed in LSCC, but its specific mechanism of action in LSCC is unknown. In this study, we aimed to investigate the effects of circYARS on the biological functions of LSCC cells. Here, with the help of multiple experimental methods and bioinformatics software, we explored the interactions between circYARS and miR-29a-3p and IREB2, as well as the mechanism of action by which circYARS modulates the miR-29a-3p/IREB2 axis to promote LSCC development.

This study found that circYARS was differentially expressed in LSCC, and explored the mechanism by which circYARS absorbs miR-29a-3p and up-regulates IREB2 to accelerate LSCC progression.

## Materials and methods

### Clinical sample

A total of 58 patients with LSCC undergoing surgery in Cancer Hospital, Chinese Academy of Medical Sciences and Peking Union Medical College were included. Adjacent normal tissue was taken at a distance of 5 cm from the tumor margin. All cancerous tissues were histologically confirmed as LSCC. Fresh specimens were immediately frozen in liquid nitrogen. Inclusion criteria: (1) Newly diagnosed LSCC patients; (2) Urine test, electrocardiogram, blood test, stool scope, X-ray, and B-type ultrasound indicated the major organs in good condition; (3) No previous anti-tumor therapy; (4) Follow-up was 5 years. Exclusion criteria: (1) Recurrent LSCC; (2) Other clinical diseases; (3) History of malignant tumor. 5 year follow-up was performed on all patients, and anyone who died during follow-up or who lost was excluded. A study approval was obtained from the Ethics Committee of Cancer Hospital, Chinese Academy of Medical Sciences and Peking Union Medical College. Informed consent was given to all patients.

### Cells

Human laryngeal epidermoid carcinoma cell Hep-2, human laryngeal carcinoma cell AMC-HN-8, and normal bronchial epithelial cell 16HBE were obtained from ATCC (Virginia, USA), and human laryngeal squamous cell TU177 and laryngeal carcinoma cell TU686 were from the National Collection of Authenticated Cell Cultures (Shanghai, China). Hep-2, TU177, TU686, and 16HBE were detected in RPMI-1640 (ATCC, Virginia, USA) in an incubator (Thermo Fisher Scientific). AMC-HN-8 was cultured in DMEM (ATCC, Virginia, USA). All cell culture media were supplemented with 10% Fetal Bovine Serum (FBS, Gibco, Grand Island, NY, USA) and 1% penicillin/streptomycin (Beyotime, Shanghai, China). Cell culture was maintained at 37 ℃ and 5% CO_2._

### Cell transfection

circYARS siRNA (si-circYARS), IREB2-overexpressing plasmid (pcDNA3.1-IREB2), miR-29a-3p-mimic, miR-29a-3p-inhibitor, and negative controls (si-NC, pcDNA3.1-NC, mimic-NC, and inhibitor-NC) were produced by GenePharma (Shanghai, China). When the cell fusion reached 60–70%, the above oligonucleotides and plasmids were transfected into TU177 cells instantaneously based on Lipofectamine 3000 (Invitrogen, Carlsbad, CA, USA). Cells were collected 24 h later and transfection efficiency was evaluated using RT-qPCR or Western blot.

### RNase R treatment

Total RNA (2 μg) of TU177 cells was extracted and digested for 10 min at 37 ℃ with or without the addition of 3 μg RNAse R (Epicentre, USA). RNA was purified by the RNeasy MinElute Cleanup kit (Qiagen, Duesseldorf, Germany), followed by RT-qPCR to detect circYARS and linear YARS.

### Actinomycin D (Act D) experiment

To test the stability of circYARS and its linear isoforms, TU177 cell whole culture medium was added with 5 µg/ml ActD (Sigma, St. Louis, MO, USA). The extracted RNA was collected at 0 h, 3 h, 6 h and 12 h, detected by RT-qPCR using HiScript II first strand cDNA synthesis kit (Vazyme, Nanjing, China), and normalized to 0 h measurements.

### RT-qPCR

Tissues and cells were harvested using TRIzol reagent (Tiangen, Beijing, China) for total RNA analysis. NanoDrop-2000 (Thermo Fisher Scientific, Waltham, MA, USA) was then applied for quantification. For mRNA and circRNA, reverse transcription was conducted using HiScript II first strand cDNA synthesis kit (Vazyme, Nanjing, China). cDNA of miRNA was produced using miRNA reverse transcription kit (TaKaRa, Tokyo, Japan). RT-qPCR was then done using SYBR-Green Real-time PCR Master Mix (Toyobo, Osaka, Japan) in the ABI PRISM 7900 system (Applied Biosystems, Shanghai, China). All primers (Table 1) were synthesized from RiboBio (Guangzhou, China). U6 was the internal parameter of miR-29a-3p, and GAPDH was that of circYARS, YARS, and IREB2. Genes were calculated by 2^−ΔΔCt^.

**Table 1:** Primer sequences

Name	Primer sequences (5′-3′)
circYARS	Forward: GGCATCTCAGTCAAATCCCC
	Reverse: ACAGTCCGTGGTTCAGCTT
YARS	Forward:TGCACCTTATCACCCGGAAC
	Reverse:TGGGCACAAAGTAAGCCACA
miR-29a-3p	Forward: CTGGTGTCGTGGAATTCAGTTGA
	Reverse:CCTGGCTCCTCACTTGGC
IREB2	Forward: AAGTCCATCCTGCTTGTCCG
	Reverse:TGCAGGGAAGCTTCTTAGGC
GAPDH	Forward: GAAGGTCGGAGTCAACGGATT
	Reverse: CTTCCCGTTCTCAGCCTTGA
U6	Forward: CTCGCTTCGGCAGCACA
	Reverse: AACGCTTCACGAATTTGCGT

### Luciferase reporter gene assay

In the starBase 3.0 (http://starbase.sysu.edu.cn/), potential binding sites shared by miR-29a-3p and circYARS or IREB2 3′UTR were predicted, respectively. GenePharma synthesized wild-type and mutant circYARS and IREB2 fragments containing miR-29a-3p binding sites. Wild-type products (circYARS-WT and IREB2-WT) and mutant ones (circYARS-MUT and IREB2-MUT) were cloned into pmirGLO luciferase reporters (Promega, Madison, WI, USA), respectively. TU177 cells were placed in 96-well plates with 1 × 10^4^ cells per well. Lipofectamine^®^3000 (Invitrogen, Carlsbad, CA, USA) was utilized to transfect luciferase reporters and miR-29a-3p-mimic or mimic-NC into TU177 cells and cultured at 37 ℃ in 5% CO_2_ for 48 h. Using a dual luciferase reporter gene assay system (Promega, Madison, WI, USA), luciferase activity was measured with Synergy 2 Multidetector Microplate Reader (BioTek Instruments Inc., Biotek Winooski, Vermont, USA).

### RIP

TU177 cells were lysed with complete RIP lysis buffer (Millipore, USA). The supernatant was then transferred to a nuclease-free test tube, and magnetic beads bound to Ago2 (#ab32381, Abcam, Cambridge, MA, USA) or IgG (#02-6102, Invitrogen, Carlsbad, CA, USA) were co-incubated at 4 ℃ for 6 h. After eluting the immunoprecipitate of the bound bead with eluting buffer, the RNA samples were purified and analyzed by RT-qPCR.

### CCK-8 experiment

TU177 cells were transfected for 24, 48, and 72 h and combined with 10 μL CCK-8 (Beyotime, Shanghai, China). A microplate reader (Bio-Rad, Hercules, CA, USA) was taken to record the optical density (OD_450nm_) after incubation in a thermostatic incubator (ThermoFisher Scientific, Waltham, MA, USA) for 1 h.

### Flow cytometry

Annexin V-FITC/PI apoptosis detection kit (Immunotech, Marseille, France) was taken for cell apoptosis detection. A cell suspension of about 200 µl at 1 × 10^6^ cells/ml was re-suspended and washed twice with pre-cooled PBS. Cells were resuspended with 500 µl in 1 × binding buffer and stained with 5 µl Annexin V-FITC/PI in the dark, respectively. After dark incubation at room temperature for 15 min, cells were loaded on a FACScan flow cytometer (Becton Dickinson, Franklin Lakes, NJ, USA) for apoptosis analysis.

### Colony formation experiment

TU177 cells (1 × 10^3^ cells/well) were put into a 6-well plate containing 10% FBS-RPMI-1640 and cultured in a 37 ℃ incubator for 14 days, during which the medium was changed every 5 days. When the petri dish formed into colony balls, the culture was stopped, and cells were cleaned with PBS. Fixed with 4% formaldehyde (Beyotime, Shanghai, China), cells were detected with crystal violet (Beyotime, Shanghai, China) for 15 min, and the colony formation rate was observed and calculated.

### Transwell experiments

TU177 cells were incubated with serum-free RPMI-1640 for 12 h prior to Transwell assay. Matrigel (Corning Incorporated, Corning, NY, USA) was stored overnight at 4 °C. While no matrix gel was used in the migration experiments. Transwell chambers were placed separately in 24-well plates. Each lower chamber was filled with 500 µl 20% FBS-RPMI-1640. Meanwhile, cells diluted with serum-free RPMI-1640 were plated in each upper chamber at 1 × 10^5^ cells/100 μl/well. The upper chamber coated with 10 µl Matrigel was taken for invasion test. After 48 h, Transwell chambers were placed into a new 24-well plate containing 4% paraformaldehyde (Beyotime, Shanghai, China). After fixation for 5 min, invaded and migrated cells were stained with 0.5% crystal violet (Beyotime, Shanghai, China) for 10 min and counted in 5 visual fields.

### Western blotting

Cells and tumor tissues were washed twice with pre-cooled PBS. Total protein was extracted using pre-cooled RIPA lysis buffer (Solarbio, Beijing, China). The protein concentration was determined using the BCA kit (Thermo Fisher Scientific, Waltham, MA, USA) and adjusted with the loading buffer (Beyotime, Shanghai, China). Proteins of equal concentration were added to the prepared 12% SDS-PAGE gel (Beyotime, Shanghai, China) and run on an electrophoretic apparatus (Bio-Rad, Hercules, CA, USA) at 80 V for 40 min and at 120 V for 2 h. PVDF protein membrane (Beyotime, Shanghai, China) was ice-bathed at 200 mA for 2 h, sealed with TBST (Beyotime, Shanghai, China) containing 5% skim milk powder for 1 h, and inoculated with the primary antibody IREB2 (ab232994, Abcam, Cambridge, MA, USA), Vimentin (ab92547, Abcam, Cambridge, MA, USA), E-cadherin (14472, Cell Signaling Technology, Danvers, MA,USA), N-cadherin (13116, Cell Signaling Technology, Danvers, MA, USA), GADPH (5174, Cell Signaling Technology, Danvers, MA,USA) were combined and incubated at 4 °C overnight. The imprinted membrane was incubated with the secondary anti-IgG (ab124055, Abcam, Cambridge, MA, USA) for 2 h, washed by TBST 3 times, and developed with ECL solution (Beyotime, Shanghai, China) in a chemiluminescence imager (Image Quant LAS4000 mini, GE Healthcare, Little Chalfont, Buckinghamshire, UK). The grayscale values of the protein bands were analyzed by Image J software. The relative expression of IREB2, Vimentin, E-cadherin, and N-cadherin in the samples was calculated based on the ratio of the gray scale values of the target protein bands to the gray scale values of the GADPH bands.

### Tumor xenotransplantation

Animal experiments were approved by the Animal Committee of Cancer Hospital, Chinese Academy of Medical Sciences and Peking Union Medical College. TU177 cells (1 × 10^6^ cells/mL) transfected with si-circYARS or si-NC were reconstituted with 100 μl PBS(Beyotime, Shanghai, China). Four-week-old female BALB/c nude mice (Shanghai Lab. Animal Research Center, China), 5 mice per group, were given an injection of the prepared cell suspension at the right flank. Three times every week, the transplanted tumor’s diameter was measured using a caliper. The mice were euthanized by cervical dislocation at week 4 after the final measurement. The tumors were weighed and photographed. The tumors were fixed with 10% formalin (Sigma, St. Louis, MO, USA) and then embedded with paraffin wax. IREB2 levels were detected by immunohistochemistry. Tumor volume = 0.5 × length × width^2^.

### Immunohistochemical (IHC) staining

The tumors embedded in paraffin were cut into 5 μm slices using microtome (RM2016, leicaSOLMS, Germany) and dewaxed with xylene (Beyotime, Shanghai, China). After blocking endogenous peroxidase activity by 0.3% H_2_O_2_ (Sigma, St. Louis, MO, USA) for 10 min, the slices were subjected to nonspecific binding to PBS solution containing 5% FBS and 0.3% Triton X-100 (Beyotime, Shanghai, China) for 1 h and combined with IREB2 (ab85051, Abcam, Cambridge, MA, USA) at 4 °C overnight. After IgG incubation (ab124055, Abcam, Cambridge, MA, USA) for 1 h, the target signal was stimulated by DAB substrate (Vector Labs, Burlingame, CA, USA), and the slices were further stained with hematoxylin for 2 min and observed with a microscope(Leica).

### Statistical analysis

All data were analyzed using Graphpad Prism 8 and expressed as mean ± standard deviation (SD). All experiments were biologically replicated at least three times (n = 3). The expression of circYARS, miR-29a-3p and IREB2 in LSCC tissues and adjacent normal tissues were compared using paired t-test. Student's t-test evaluated bilateral comparisons, while one-way ANOVA evaluated multiple comparisons. *p < 0.05 was considered statistically significant.

## Results

### circYARS is upregulated in LSCC and is associated with shorter survival

**Fig. 1:**
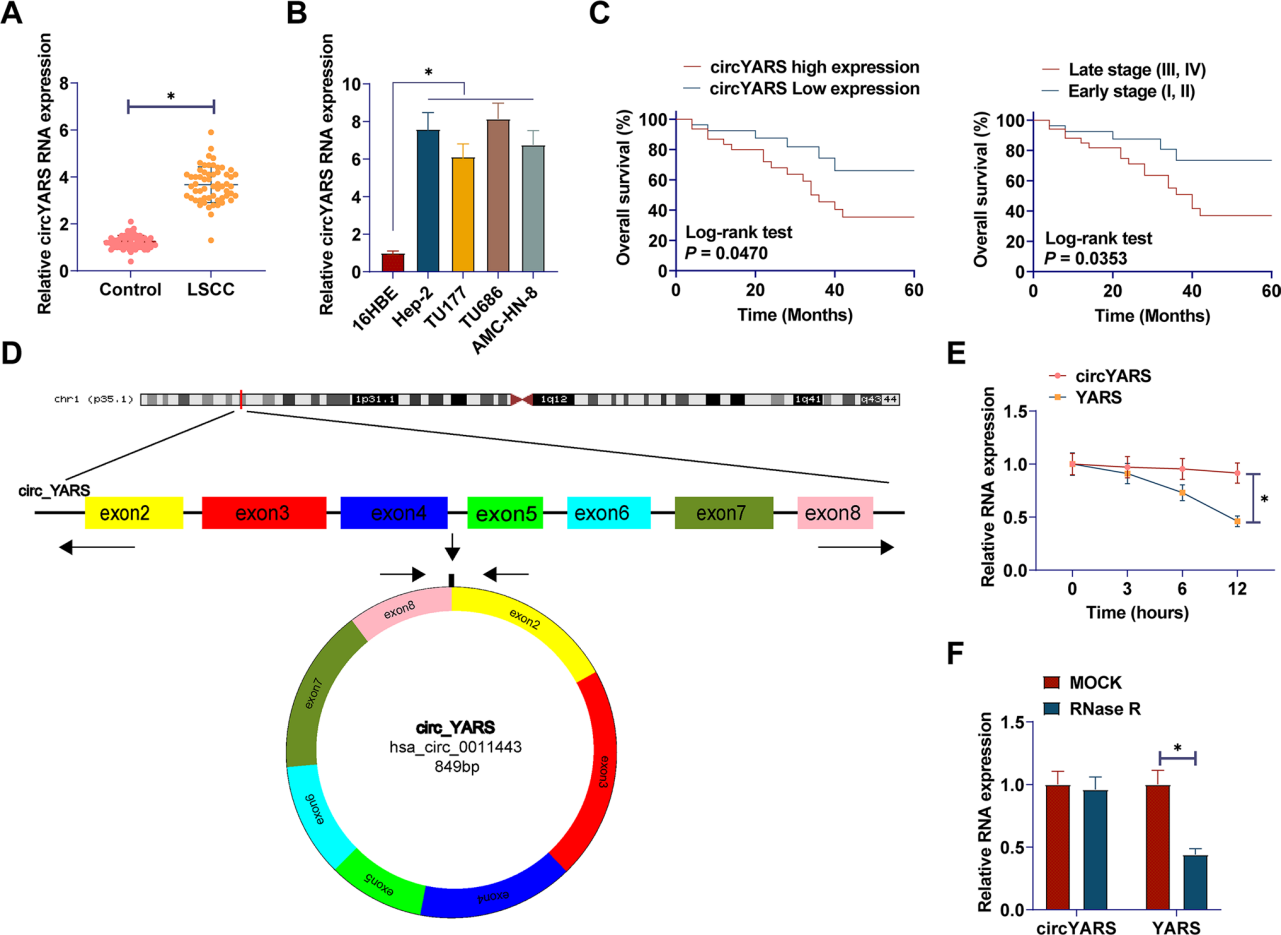
circYARS is upregulated in LSCC and is associated with shorter survival time. **A**, **B** circYARS levels in LSCC tissues and cells; **C**: Kaplan–Meier analysis of circYARS expression and survival. **D** circYARS structure; **E**: circYARS expression after ActD treatment; **F**: Stability of circYARS after RNAse R treatment. Data are expressed as mean ± SD (n = 3). Paired t-tests and student t-tests were used. *P < 0.01

circYARS in 58 pairs of LSCC tissues and non-tumor tissues was preliminarily analyzed by RT-qPCR. Significantly increased circYARS expression levels were found in LSCC tissues compared to non-tumor tissues (Fig. 1A). circYARS in four LSCC cell lines and 16HBE was shown in (Fig. 1B), and circYARS in LSCC cells was significantly increased. Kaplan–Meier analysis showed that LSCC patients with high circYARS expression had shorter overall survival (Fig. 1C). It was confirmed that high circYARS expression was correlated with TNM staging and T classification of LSCC patients. However, there was no correlation with age, gender, or tumor size (Table 2). The structure of circYARS is shown in the figure (Fig. 1D). circYARS were treated with 5 µg/ml ActD at 0 h, 3 h, 6 h, and 12 h, and circYARS were not sensitive to Act D (Fig. 1E). At the same time, circYARS treated with RNase R did not change significantly (Fig. 1F), indicating that circYARS had a stable ring structure. Taken together, the results suggest that circYARS is a stable circular RNA with up-regulated expression in LSCC.

**Table 2:** Correlation between circYARS expression and clinical variables in LSCC patients

Parameters	N	circYARS low expression (n = 29)	circYARS high expression (n = 29)	P value
Gender				
Male	36	19	17	0.7871
Female	22	10	12	
Age (years)				
≤ 60	33	17	16	0.7909
> 60	25	12	13	
Smoking history				
No	17	9	8	0.773
Yes	41	20	21	
T category				
T1‐T2	36	22	14	0.0304
T3‐T4	22	7	15	
TNM stage				
I‐II	10	8	2	0.037
III‐IV	48	21	27	
Tumour differentiation				
Well to moderate	34	20	14	0.1097
Poor	24	9	15	

### circYARS enhances malignancy of LSCC cells

**Fig. 2:**
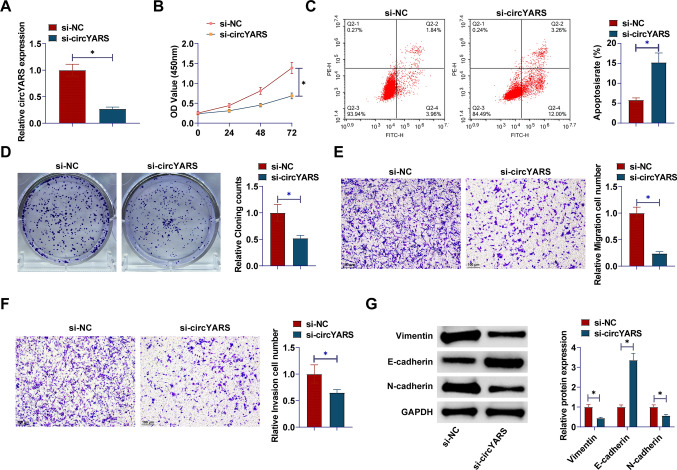
circYARS accelerates malignancy of LSCC cells. **A**: circYARS expression after interference; **B**: CCK-8 assay detection of cell proliferation; **C**: Flow cytometry analysis of apoptosis rate; **D** Colony formation assay of colony-forming ability; **E**, **F**: Transwell detection of cell invasion and migration ability; **G**: Western blot analysis of Vimentin, E-cadherin, and N-cadherin. Data are expressed as mean ± SD (n = 3). Student t-tests were used. *P < 0.05

To investigate the potential function of circYARS in LSCC, si-circYARS was designed to interfere with TU177 cells. circYARS expression in TU177 cells was decreased by si-circYARS, indicating significant transfection efficiency of si-circYARS (Fig. 2A). CCK-8 assay and flow cytometry suggested that circYARS silencing effectively reduced cell proliferation capacity and promoted apoptosis in TU177 cells (Fig. 2B, C). To further evaluate cell proliferation capacity, colony formation assay was performed, and TU177 cell colony formation decreased after circYARS silencing (Fig. 2D). In Transwell assays, TU177 cell migration and invasion decreased after circYARS silencing (Fig. 2E, F). The effect of circYARS silencing on EMT-related proteins was assessed by Western blotting analysis. Downregulating circYARS increased E-cadherin and reduced Vimentin and N-cadherin proteins (Fig. 2G).

### circYARS directly interacts with miR-29a-3p

**Fig. 3:**
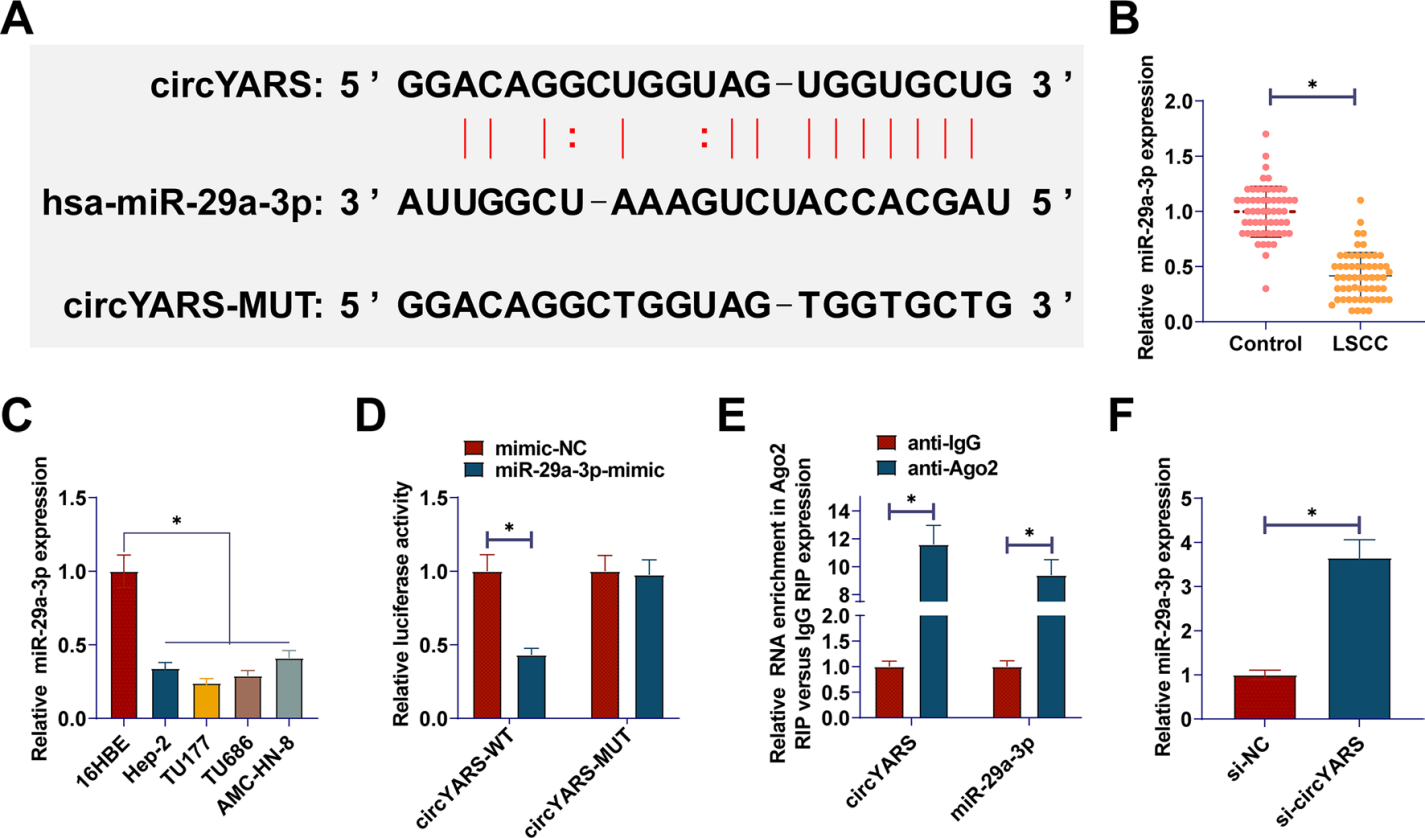
circYARS directly interacts with miR-29a-3p. **A**: Bioinformatics prediction of target binding sites between circYARS and miR-29a-3p; **B**, **C**: miR-29a-3p levels in LSCC tissues and cells. **D**, **E**: Investigation of the interaction between miR-29a-3p and circYARS;** F** miR-29a-3p expression change after circYARS interference. Data are expressed as mean ± SD (n = 3). Paired t-tests and student t-tests were used. *P < 0.01

The Starbase showed the binding site of circYARS to miR-29a-3p (Fig. 3A), confirming miR-29a-3p as a direct target of circYARS. RT-qPCR results indicated miR-29a-3p downregulation in LSCC tissues and cells (Fig. 3B, C). Dual luciferase reporter gene experiment observed that miR-29a-3p mimic and circYARS-WT cotransfection decreased luciferase activity. miR-29a-3p mimic and circYARS-Mut cotransfection showed no significant change in luciferase activity (Fig. 3D). Meanwhile, it was demonstrated in the RIP experiment that Ago2 immunoprecipitated circYARS and miR-29a-3p, suggesting that miR-29a-3p is a target of circYARS in TU177 cells (Fig. 3E). After transfecting si-circYARS into TU177 cells, RT-qPCR results showed that knockdown of circPDK1 enhanced miR-4731-5p expression (Fig. 3F).

### miR-29a-3p downregulation mitigates si-circYARS-mediated inhibition of malignant behavior in LSCC cells

**Fig. 4:**
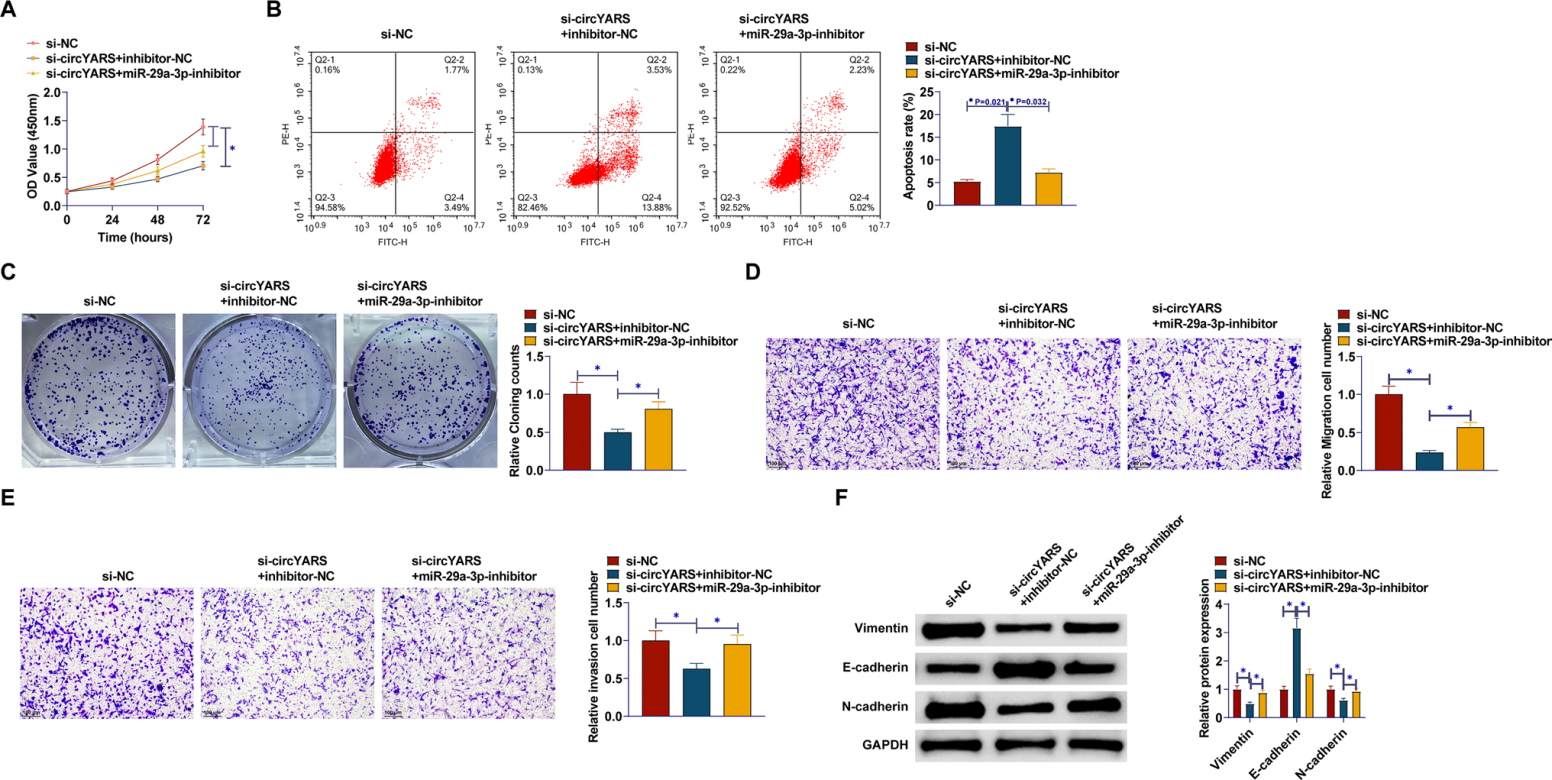
Depleting miR-29a-3p mitigates si-circYARS mediated inhibition of malignant behavior of LSCC cells. **A**: CCK-8 assay detection of cell proliferation; **B**: Flow cytometry analysis of apoptosis rate; **C** Colony formation assay of colony-forming ability; **D**, **E**: Transwell detection of cell invasion and migration ability; **F**: Western blot analysis of Vimentin, E-cadherin, and N-cadherin. Data are expressed as mean ± SD (n = 3). Comparisons between the three groups were made using the one-way ANOVA test. *P < 0.05

A series of rescue experiments were conducted to verify the role of circYARS and miR-29a-3p in LSCC. CCK-8 assay observed that si-circYARS-induced inhibition of TU177 cell proliferation was mitigated by miR-29a-3p inhibitor (Fig. 4A). Flow cytometry results noted that miR-29a-3p inhibitor mitigated the apoptosis-promoting effect of si-circYARS (Fig. 4B). Colony formation experiment proved that si-circYARS reduced the formation of cancer cell colonies, but downregulating miR-29a-3p increased the number of colonies formed (Fig. 4C). Transwell assays suggested that si-circYARS limited cell migration and invasion, and this effect was alleviated after depleting miR-29a-3p (Fig. 4D, E). Western blotting demonstrated that si-circYARS increased E-cadherin factor and decreased Vimentin and N-cadherin, but suppressing miR-29a-3p mitigated the influence of circYARS silencing on the above EMT factors (Fig. 4F).

### circYARS up-regulates IREB2 by targeting miR-29a-3p

**Fig. 5:**
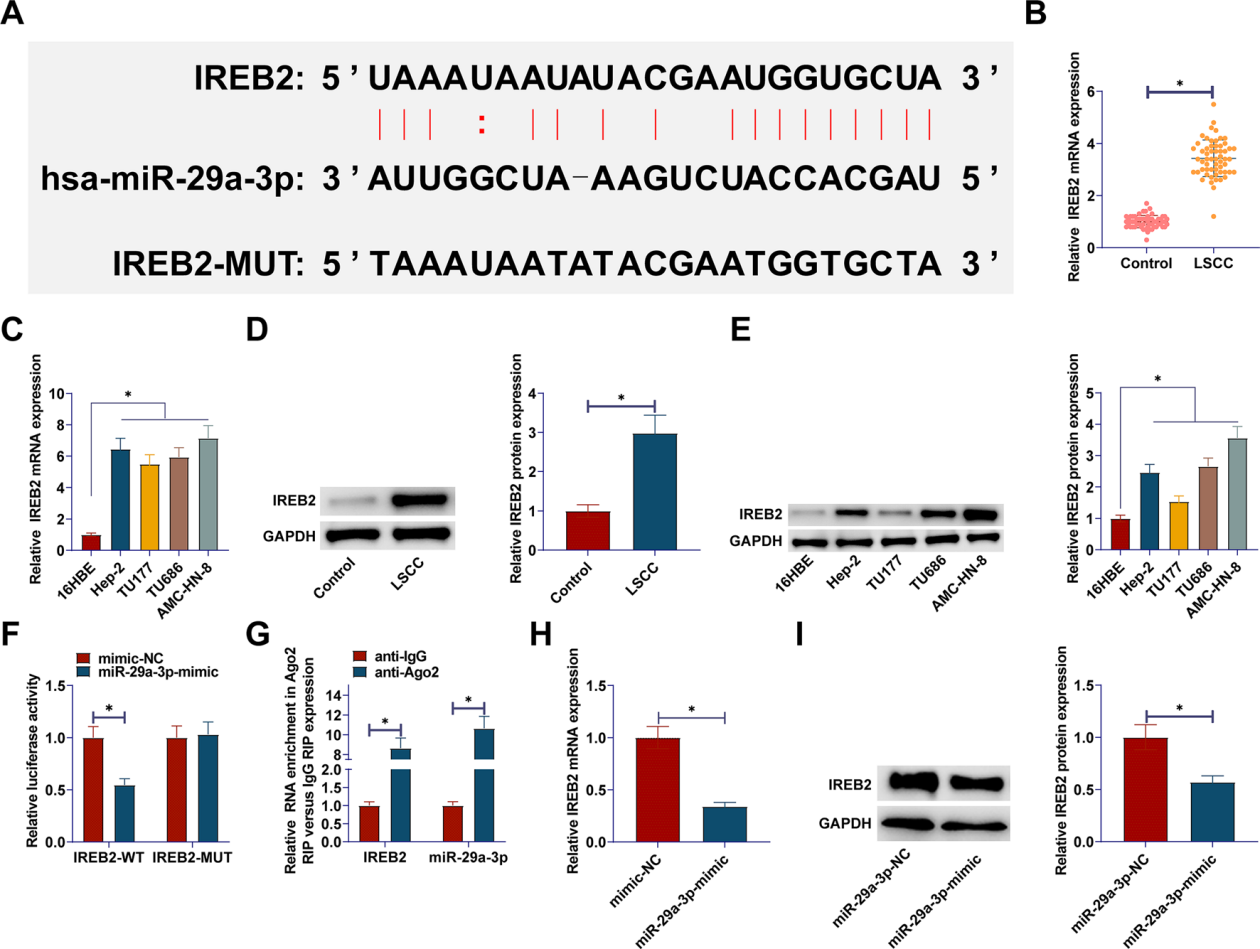
circYARS up-regulates IREB2 by targeting miR-29a-3p. **A**: Bioinformatics prediction of target binding sites between IREB2 and miR-29a-3p; **B**–**E**: miR-29a-3p levels in LSCC tissues and cells. **F**, **G**: Investigation of the interaction between IREB2 and miR-29a-3p; **H**, **I**: IREB2 expression change after miR-29a-3p interference. Data are expressed as mean ± SD (n = 3). Comparisons between two groups were made using paired t-tests and student t-tests; comparisons between multiple groups were made using ONE-WAY ANOVA tests. *P < 0.01

Complementary sites between the IREB2 3 'UTR and miR-29a-3p sequences are shown (Fig. 5A). IREB2 gene and protein were elevated in LSCC tissues and cells (Fig. 5B, E). Both luciferase reporter assay and RIP experiment confirmed the fact that miR-29a-3p targets IREB2 (Fig. 5F, G). miR-29a-3p-mimic was transfected into TU177 cells, leading to inhibition of IREB2 gene and protein levels (Fig. 5H, I).

### IREB2 overexpression mitigates si-circYARS-mediated inhibition of malignant behavior in LSCC cells

**Fig. 6:**
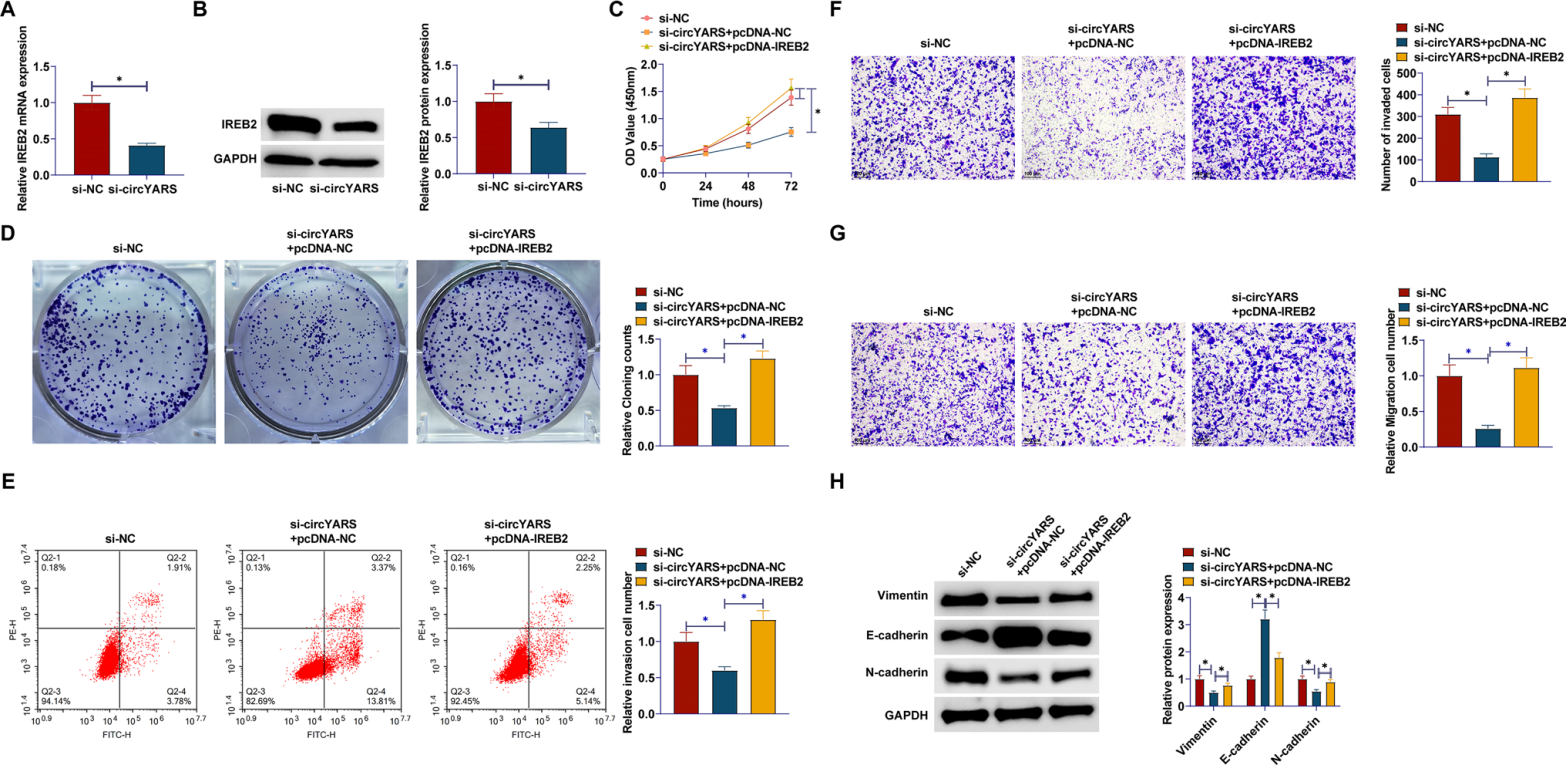
IREB2 overexpression mitigates si-circYARS-mediated inhibition of malignant behavior in LSCC cells. **A**, **B**: IREB2 expression after interference; **C**: CCK-8 assay detection of cell proliferation; **D**: Colony formation assay of colony-forming ability; **E**: Flow cytometry analysis of apoptosis rate; **F**, **G**: Transwell detection of cell invasion and migration ability; **H**: Western blot analysis of Vimentin, E-cadherin, and N-cadherin. Data are expressed as mean ± SD (n = 3). Comparisons between two groups were made using the student t-test; comparisons between multiple groups were made using the one-way ANOVA test. *P < 0.05

In TU177 cells, si-circYARS simultaneously down-regulated IREB2 (Fig. 6A, B). To further explore whether circYARS can regulate IREB2 expression by binding to miR-29a-3p in LSCC cells, a series of rescue experiments were performed. si-circYARS inhibition of TU177 cell proliferation was mitigated by overexpressing IREB2 (Fig. 6C, D). Upregulating IREB2 saved the apoptosis-promoting effect of si-circYARS (Fig. 6E). si-circYARS limited cell migration and invasion, and this effect was rescued after overexpressing IREB2 (Fig. 6F, G). Vimentin and N-cadherin proteins increased and E-cadherin protein levels reduced in cells transfected with si-circYARS and pcDNA3.1-IREB2, compared with cells transfected with si-circYARS only (Fig. 6H).

### circYARS enhances the growth of LSCC cell xenografts in vivo

**Fig. 7:**
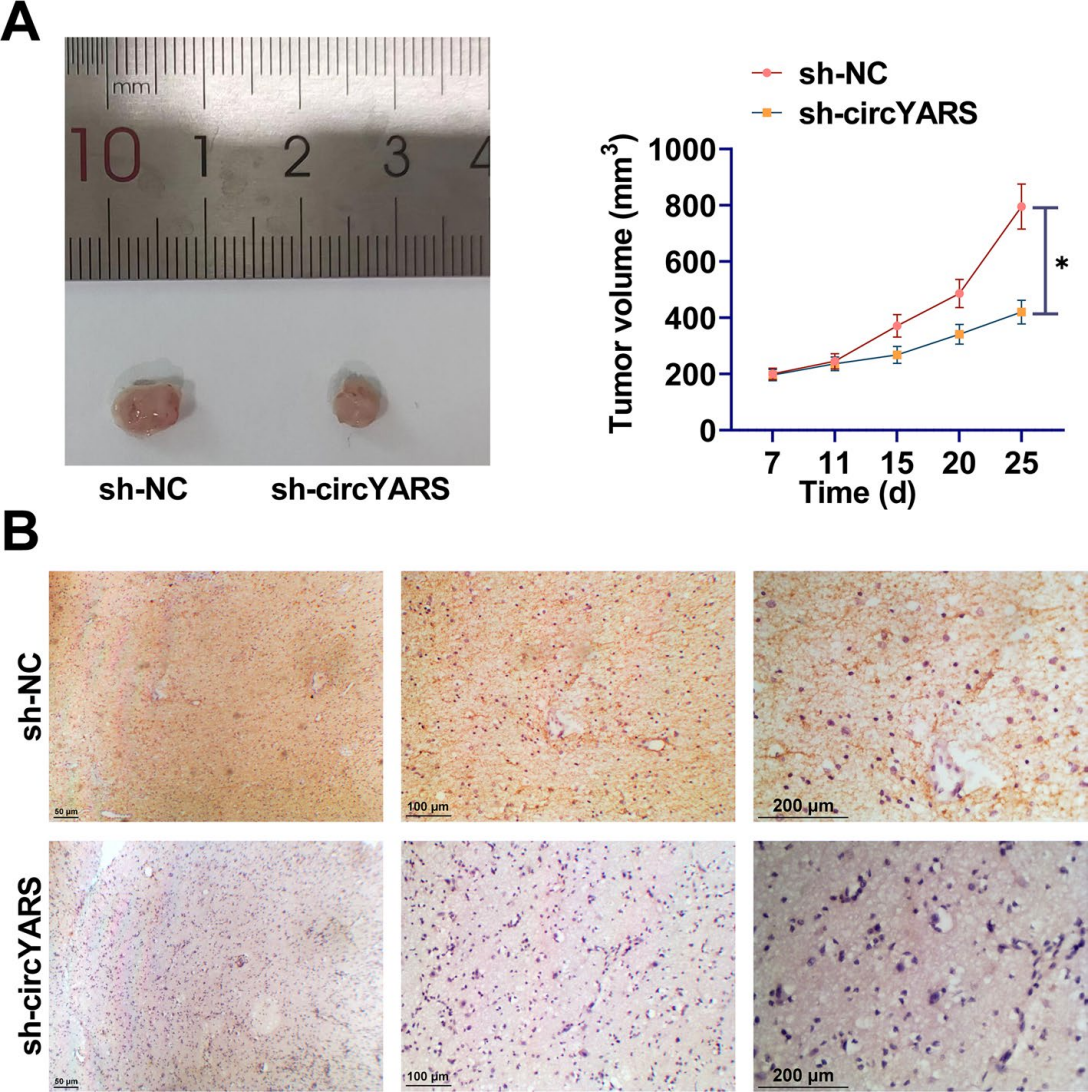
circYARS enhances the growth of LSCC xenografts in vivo*.* **A**: Tumor volume; **B**: IHC staining of IREB2 expression in xenograft tumors. Data are expressed as mean ± SD (n = 5). Comparisons between the two groups were made using paired t-tests and student t-tests. *P < 0.05

TU177 cells were transfected with sh-circYARS or sh-NC and then inoculated into mice. The mean tumor volume in the si-circYARS group were smaller than in the si-NC group (Fig. 7A). IHC staining of xenograft tumors showed that IREB2 expression was reduced by circYARS knockdown (Fig. 7B).

## Discussion

Over the past few decades, LSCC patients' overall survival times have not improved significantly [24]. In addition to regulating gene transcription and translation, circRNAs also regulate protein interactions and miRNA sponges [25, 26]. The discovery of new therapeutic targets and diagnostic biomarkers for cancer may be made possible by circRNAs [27].

Differential expression of circRNAs has been revealed in LSCC [28, 29]. A novel circRNA was also identified in the study, as well as an overexpression of circYARS in LSCC that was preliminarily confirmed. High expression of circYARS was correlated with TNM staging of LSCC. At the same time, interference with circYARS reduced malignant behaviors of LSCC cells. Vimentin, E-cadherin, and N-cadherin are markers associated with EMT to indicate tumor development. As expected, circYARS knockdown decreased Vimentin and N-cadherin and increased E-cadherin in LSCC cells. This suggested that circYARS knockdown inhibited EMT characteristics of LSCC cells, suggesting that circYARS is an important oncogene that promotes the malignant progression of LSCC. This is consistent with previous studies on the mechanism of action of other circRNAs on LSCC. Knockdown of circRNAs inhibits the proliferation, invasion and migration of LSCC cells, and promotes apoptosis, thus inhibiting the onset and progression of LSCC. For example, the circRASSF2/miR-302b-3p/IGF-1R axis promotes the proliferation and invasion of LSCC cells [30].

CircRNAs have been thought to act as ceRNA to absorb miRNAs and regulate their downstream targets [31]. The interaction between miRNA and circRNA can regulate the malignancy and aggressiveness of cancer cells [32, 33]. circSLC7A11 promotes LSCC cell progression and stemness by absorbing miR-877-5p [34]. Hsa_circ_0042823 triggers LSCC progression through downregulating miR-877-5p [35]. To further investigate the biological function of circRNA_103862, its ceRNA mechanism in LSCC was analyzed. This research indicated that miR-29a-3p level was low in LSCC cells, and circYARS and miR-29a-3p interacted to regulate the malignant characteristics of LSCC cells. Based on these data, it was concluded that the oncogenic effect of circRNA_103862 depends on its inhibition of miR-493-5p.

Iron mutation-associated genes are responsible for regulating tumor cell death through iron-dependent regulation, driven by the accumulation of lipid peroxides. Induction of ferroptosis has become a promising therapeutic approach [36]. Ferroptosis may be closely associated with LSCC tumor progression through KEGG pathway analysis [37]. Importantly, 50% of ferroptosis-related genes were differentially expressed between LSCC tumor tissues and normal samples, suggesting that ferroptosis-related genes could be used as biomarkers to predict LSCC. In this study, IREB2 was proved to be miR-29a-3p’s target gene, and circYARS can sponge miR-29a-3p to enhance IREB2, thus accelerating LSCC progression. IREB2 expression was upregulated in LSCC tissues. Elevated levels of IREB2, a major transcription factor for iron metabolism, lead to iron accumulation and lipid peroxidation, which are essential for triggering ferroptosis. The underlying mechanisms of LSCC tumorigenesis have been a hot topic, but how ferroptosis-related genes regulate tumor progression remains poorly understood. Currently, we are not sure whether IREB2 regulates LSCC progression through ferroptosis. In the future, we will continue to explore the mechanism of action of IREB2 and ferroptosis in LSCC with the aim of providing new insights into the therapeutic mechanism of LSCC and new therapeutic targets and strategies.

Despite these findings, the study has some limitations.CircYARS functions through a ceRNA network, and we have only observed its significance for the relation between circYARS/miR-29a-3p/IREB2. To better understand circYARS in LSCC, other miRNAs and mRNAs must be investigated. These findings should be validated in a large number of clinical samples to increase credibility.

## Conclusion

circYARS/miR-29a-3p/IREB2 axis regulates LSCC progression. Results from this study shed light on circYARS' treatment of LSCC and circYARS may be a promising biomarker for future studies of the diagnosis and prognosis of LSCC.

## Data Availability

The datasets used and/or analyzed during the present study are available from the corresponding author on reasonable request.

## Declarations

### Tumor size in experimental animals

The maximum tumour size/burden permitted by the Ethics Committee is a volume of 2000 mm^3^. No mouse in this study has violated this standard and did not exceed the maximum tumour size/burden.
